# Metaverse as a possible tool for reshaping schema modes in treating personality disorders

**DOI:** 10.3389/fpsyg.2022.1010971

**Published:** 2022-10-10

**Authors:** Bin Yin, Ya-Xin Wang, Cheng-Yang Fei, Ke Jiang

**Affiliations:** ^1^Laboratory for Learning and Behavioral Sciences, School of Psychology, Fujian Normal University, Fuzhou, Fujian, China; ^2^Department of Applied Psychology, School of Psychology, Fujian Normal University, Fuzhou, Fujian, China; ^3^School of Mental Health, Wenzhou Medical University, Wenzhou, Zhejiang, China

**Keywords:** metaverse, personality disorders, schema therapy, Piaget, genetic epistemology, digital mental health services, experience setting, mental health care

## Abstract

Personality disorders (PD) are usually treated with face-to-face sessions and/or digital mental health services. Among many schools of therapies, schema therapy stands out because rather than simply targeting the symptoms of PD, it cordially targets the cause of PD and heals the early maladaptive schema, thus is exceptionally good at soothing emotional disturbances before enacting cognitive restructuring, resulting in long-term efficacy. However, according to Piaget’s genetic epistemology, the unmet needs lie in the fact that the schemata that determine the adaptive behavior can only be formed in the interaction with the real world that the patient is living in and reconsolidated by the feedback from the object world upon the patient’s newly-formed behavior. Therefore, in order to reshape the patient’s schema modes to support adaptive behavior and regain emotional regulation capabilities of the healthy adult, one may have to reconstruct the object world surrounding the patient. Metaverse, the bestowed successor to the Internet with the cardinal feature of “the sense of full presence,” can become a powerful tool to reconstruct a new object world for the patient with the prescription of a psychotherapist, so as to transform the treatment techniques in schema therapy into the natural autobiographical experiences of patients in the new object world, thus gradually reshape the patient’s schema modes that can ultimately result in an adaptive, and more inclusive, interaction with the real world. This work describes the underlying theory, the mechanism, the process, and ethical considerations of such promising technology for the not-too-far future.

## Introduction: Personality disorders and schema therapy

Personality disorders (PD) refer to “an enduring pattern of inner experience and behavior that deviates from the expectations of the individual’s culture” (American Psychiatric Association, 2013), including several subtypes, such as borderline personality disorder (BPD), narcissistic personality disorder (NPD), paranoid personality disorder (PPD), avoidant personality disorder (APD), antisocial personality disorder (ASPD), schizotypal personality disorder (SPD), etc. People with PD are generally difficult to cope with, have unrelenting relationship problems with emotional dysregulation, and may cause harm to either people in his/her surroundings or him/herself and even commit crimes (Widiger and Costa Jr, 1994; Zachar et al., 2016; Dadomo et al., 2018). Indeed, even not diagnosed, we can observe in our daily life “enduring,” “pervasive” and “inflexible” traits in people with “characteristic issues” (Wright and Simms, 2016), who may or may not seek help from mental health practitioners depending on the severity of their problems in their work and life, their realization of such needs weighed against perceived stigma, and the cost and accessibility to such services (Kazdin and Rabbitt, 2013; Andrade et al., 2014). As a matter of fact, even though mental health problems are widespread all over the world (Patel and Prince, 2010), studies have pointed out that even in developed countries like the United States 62% of adults with mental illness and 41% of adults with serious mental illness have not received any mental health care in the previous year (Walker et al., 2015). The COVID-19 pandemic has exacerbated this situation and urged changes in how we practice mental health care (Moreno et al., 2020; Peng et al., 2020; Vadivel et al., 2021). Therefore, it is time to consider digital mental health services (DMHS) and expand the coverage and accessibility of mental health care (Kazdin and Rabbitt, 2013; Lattie et al., 2022), especially for people with plausible PD syndromes because their symptoms are chronic or even life-time, but their motivation and approach to engage in treatment are complicated (Young et al., 2006).

Among all kinds of psychotherapeutic treatments for PD (Dixon-Gordon et al., 2011), schema therapy (ST) stands out because rather than simply targeting the symptoms of PD, it cordially targets the cause of PD and heals the early maladaptive schema (EMS), thus is exceptionally good at soothing the emotional disturbances before enacting cognitive restructuring, resulting in long-term efficacy (Young, 1999; Young et al., 2006; Dadomo et al., 2016, 2018). The term “schema” was annotated by famous psychologists from Bartlett and Piaget to Beck and Young (Pace, 1988; Adekoya, 2013). Within cognitive psychology, a schema is the developmental norm of cognitive structures (i.e., a meta-structure) formed by an individual in the process of interacting with the environment, operated by selectively organizing the on-going experience of each individual into subjectively meaningful patterns. Through the schemas, people are active constructors of their own psychological realities. Importantly, those schemas formed early in life continue to be elaborated and then superimposed on later life experiences, even when they are no longer applicable. As a result, those maladaptive schemas formed early in life (i.e., EMS) might be at the core of personality disorders, milder characterological problems, and many chronic emotional dysregulations (Young et al., 2006; Dadomo et al., 2018). The good news is that schemas not only guide behavior in response to contextual stimuli, but can also be reshaped by the interaction with the external world, thus providing a window for treatment (Dadomo et al., 2016; Taylor et al., 2017).

Indeed, schema therapists use techniques such as limited reparenting, imagery rescripting, and chair work to help clients exchange their maladaptive schema modes for an adaptive one (Dadomo et al., 2016). Accordingly, a schema mode is “an intense predominant emotional state linked to a pattern of thinking, feeling and behaving based on a set of specific needs” (Dadomo et al., 2018). These approaches have been proved to be successful in several randomized controlled trials (e.g., Taylor et al., 2013; Bamelis et al., 2014; Hoffart Lunding and Hoffart, 2016; Aloi et al., 2019; van Dijk et al., 2019). However, despite for the success of ST in treating PD, there are several lines of evidences that suggest that ST could greatly benefit from the future application of DMHS, especially the metaverse: (1) In a qualitative study done on patients’ perspective on the first phases of imagery work in the context of ST (Marieke et al., 2011), PD patients reported lacking information, communication, and support during the initial phases of imagery work, and the duration of the imagery exercises was unpredictable, which created feelings of uncertainty and fear. (2) Researchers have already tried to integrate e-health tools such as *priovi* into borderline-PD-specific treatments based on ST and showed that the integration can potentially increase treatment intensity and enhance treatment effects, though therapists should monitor the usage of e-health tools, help with difficulties, and check if patients understand them and promote their usage (Fassbinder et al., 2015). (3) In a clinical trial, Hoffart Lunding and Hoffart (2016) found that schema therapy carries the risk to lead to a more negative view of parents’ care during upbringing and this risk is accentuated with less benefit of therapy—this is because education about EMS enact patients’ awareness of such negative experiences and may become burdensome for patients to benefit from ST. All these evidence suggest that if effortful imagination or homework in the ST could be replaced by effortless experience in the metaverse, PD patients could have their maladaptive schema modes reshaped unconsciously and regain healthy schema modes to function in the real world.

## New possibility: Reshaping schema modes in the metaverse for treating PD

### Theoretical basis: *Jean Piaget’s theory of genetic epistemology*

**Figure 1:**
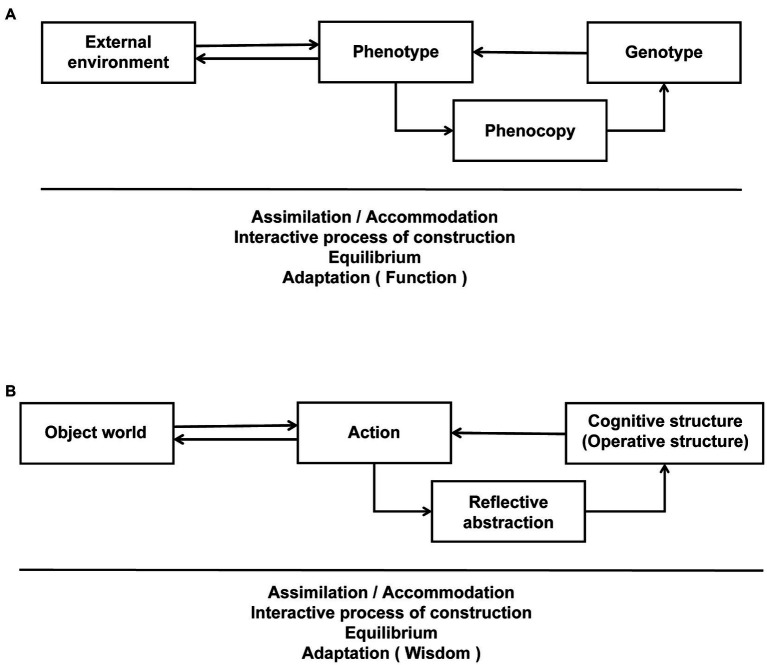
The process of interactive construction between the cognitive/operative structure and the object world (adapted and reproduced from Jiang and Li (2020) with permission). **(A)** The process of interactive construction of an organism with the external environment. The active interaction between the internal mechanisms of the organism and the external environment constructs the phenotype. There is a two-way interaction between the phenotype and the external environment as shown by the double arrows. Whereas the genotype determines the phenotype, the phenotype can feedback to the genotype through the process of phenocopy as shown by the single arrow. The effect of internal mechanisms on the external environment throughout the process is called assimilation. The active regulation of internal mechanisms is known as accommodation. Assimilation and accommodation balance the internal and external aspects of the organism to achieve an adaptive equilibrium, whose steady state is called adaptation or function. **(B)** The process of interactive construction of the cognitive/operative structure and the object world. The active interaction between the cognitive/operative structure and the object world constructs the subject’s action (behavior). The interaction between the subject’s action and the object world is shown by the double arrows. The cognitive/operative structure determines the subject’s action but receives feedback from the outcome of the action in interaction with the object world *via* reflective abstraction, as shown by the single arrow. Similarly, the effect of internal mechanisms on the external environment is referred to as assimilation, while the active regulation of the internal mechanisms is referred to as accommodation. Assimilation and accommodation allow the subject to reach equilibrium with the environment. When the environment changes and the subject is no longer able to adapt to the environment conditions, a “de-equilibration” occurs, and the need for re-equilibrium becomes a motivation for the subject to make behavioral adjustments. The individual responds to the changes in the external world through actions, and the abstracted reflection on the outcome of the new action leads to a refreshed internal cognitive/operative structure and a higher level of rebalancing. At this point, cognitive abilities also develop to a higher level. The subject is able to adapt to the environment and gain wisdom. Note that the developmental norm of the cognitive/operative structure is called a schema.

Young et al. (2006) acknowledged that in psychology the term *schema* is probably most commonly associated with Piaget, who wrote in detail about schemata in different stages of childhood cognitive development. Indeed, Piaget’s theory of *genetic epistemology* originates from his studies of epigenetics that emphasized the biological interactive construction process of both the innate mechanisms and the external environment (Figure 1A). Later he logically transferred the biological model to the field of epistemology in order to explain the constructive process of cognitive development (Figure 1B). According to this theory, the agent interacts with the object world through actions, and the reflective abstraction of the interaction, in turn, shapes the development of the cognitive/operative structure, which recursive process ultimately achieves an adaptive balance between the agent and the object world through assimilation and accommodation (Piaget, 1974/1980, 1976/1978, 1977a,b, 1979) or maladaptation in the case of PD. Therefore, one’s own interaction with the object world is more likely to establish causality and enact changes directly in the schema (the developmental norm of cognitive/operative structures) than observational learning or linguistic transmission of others’ experiences—this may be a key determinant for the long-term success of schema therapy. Critically, this theory has received both neurobiological (Vogel et al., 2018) and neurocomputational (Kumaran, 2013) support.

### Creating a new object world in the metaverse for treating PD

Since the interaction between the agent and the object world is the source of schema formation and updating, a key problem in the therapeutic process may lie in that the therapist has very little control over the patient’s object world, and therefore, the therapist cannot be sure that the patient’s schema modes they strive to repair and reconstruct during the therapeutic process can still be valid or thrive in its healthy form outside the physical therapeutic workspace—in other words, the adaptive schema mode may lack the condition for reconsolidation and thus may be unable to reach the new equilibrium with the object world whereas the maladaptive ones may be reinstated by the very harmful stimuli that had triggered the original defensive response because that was an equilibrium. Therefore, the core of the problem is how to truly enable the patient to achieve automatic reshaping and renewal of schema modes *via* positive experiential feedback from their interaction with the object world so as to achieve the new equilibrium—once this new equilibrium is achieved and consolidated, then the patient can be empowered to adaptively function in the real world. Seeking changes in the object world as the source of those maladaptive schema modes is urgent, and the emergence of the metaverse technology may provide us with this opportunity.

One cardinal feature of the metaverse (and its premature version, virtual reality) is the sense of full presence, i.e., the sense of “being there” (Pl) without perceiving the technology that generates it, and “plausibility” (Psi), which includes the fidelity of the depicted situation with prior knowledge and expectations so that participants can not only carry out their intentions but also find themselves exhibiting automatic behaviors and responses as if the events in the metaverse were real (Rovira et al., 2009; Slater et al., 2022), thus is able to create a kind of “second-life” experiences for participants (Gorini et al., 2008; Parsons, 2012; Best and Butler, 2013, 2015) as well as achieving high ecological validity for neuropsychological assessment and treatment planning (Parsons, 2012, 2015a, 2017).

Indeed, the metaverse or its premature version, i.e., virtual reality, has been proposed to be used in treating attention deficits/hyperactivity disorder (Schweitzer and Rizzo, 2022), autism spectrum disorder (Lorenzo et al., 2016; Herrero and Lorenzo, 2020; Hutson, 2022), post-traumatic stress disorder (Rizzo and Shilling, 2017; Crary, 2020), anxiety and specific phobias (Parsons, 2015b; Freeman et al., 2017; Ong et al., 2022), borderline personality disorders (Good et al., 2013), various forms of psychosis (Veling et al., 2014), as well as rehabilitating offenders (Seinfeld et al., 2018; Ticknor, 2019), improving empathetic skills (Barnes et al., 2022), cultivating prosocial behavior (Rosenberg et al., 2013), and helping overcoming personal problems in life (Slater et al., 2019), etc. These pioneering work has paved the way for the future of DMHS (Ifdil et al., 2022), utilizing various forms of virtual environment in the following three ways: (1) Exposing the participants to clinically meaningful and physically safe stimuli in the virtual environment, being it fearful stimuli for exposure therapy, inclusive social support for disadvantaged groups, or neutral but helpful information and/or tools/exercises for guided learning, assessment, and practices. (2) Providing an opportunity for self-talk in different avatars or taking different perspectives by means of virtual embodiment for problem solving and/or conflict reconciliation. (3) Creating a scripted virtual story for participants to experience and providing guided feedback to their actions in the virtual environment—this approach can be regarded as the “short” form of what we aim to propose here—“short” in that it targets at reinforcing specific behaviors in specific situations (e.g., how to act appropriately in a social gathering), whereas our “long” form targets at creating a new object world with a certain *experience setting*, which can be defined as the “culture” or “norm” of the new object world in which participants experience and interact with multiple characters and a series of events that would bring out a variety of principle-guided healthy outcomes upon their actions/behavior, gradually reshaping their schema modes.

**Figure 2:**
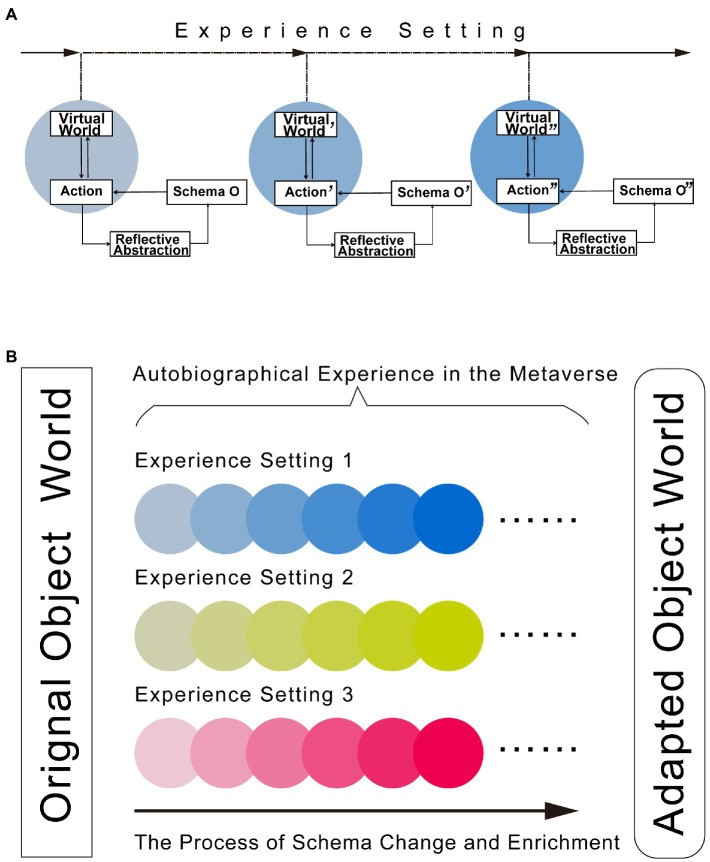
Mechanism and process of how the metaverse can be used as a tool for reshaping schema modes. **(A)** The mechanism of schema mode changes in the metaverse. After entering the metaverse, the client will encounter a virtual world with certain experience setting prescribed by the psychotherapist. The feedback of the interaction with the virtual world will be significantly different from the original world the subject is living in and where the original schema modes (O) are formed, and thus this difference in feedbacks will gradually bring about changes and enrichment in the schema modes toward a more adapted one (O′) *via* reflective abstraction. The solid arrows at the top represent entering into the metaverse and returning to the real world. The dashed arrows represent entering into the multiple stages of the virtual world with certain experience setting, which is designed to foster gradual schema mode and behavioral changes through recursive interaction and reflective abstraction. The interaction between the action of the subject and the virtual world is represented by a double arrow. The background circles with different color gradients represent the convergent changes of the virtual world as defined by the experience setting and the subject’s behavior. **(B)** Flow chart of the metaverse as a tool for reshaping schema modes and its impact on the client living in the real world. The box on the left side of the diagram represents the original object world, in which the client’s behavior pattern may be fixed and the perception of the object world is based on predetermined schema modes. Each colored circle represents the occurrence of schema mode change brought about by the interaction between the virtual world and the client’s behavior, and a series of gradual changes in the colored circles represent the gradual change and enrichment of schema modes. Through different experience settings prescribed by the psychotherapist, the subject entering the metaverse can obtain a series of autobiographical experiences that will result in a more adaptive schema mode to behave in the real object world, and the enriched schema modes can also facilitate the activation of more adapted behaviors. The various interactions between the subject and the object world will also help people shape a more inclusive and accepting environment for the client and people alike. The box on the right side of the diagram represents the adapted object world the subject is able to live in with a high degree of mental health. The arrow from left to right at the bottom represents the process of schema mode change in the subject experience the metaverse.

In other words, we aim at creating a new object world in the metaverse with a certain kind of *experience setting* that represents the world of healthy schema modes, which can bring a whole new set of experiences that are difficult for the PD patient to experience in the real world. In the real world, due to the solid balancing relationship of the old schema modes and the environment, it is difficult for the patient to perform the initial step of behavioral changes, and even if he/she tried, he/she would revert to the old action-outcome cycle due to the inappropriate feedback from the environment, which may even cause secondary harm to the patient. The new object world in the metaverse can provide a supportive and safe harbor for the patient to venture into different behaviors. The different behaviors will then receive appropriate feedbacks from the environment, which will gently bring positive and healing experiences to the patient. This process contributes to the automatic reshaping and reconsolidation of healthy schema modes (Figure 2A).

Importantly, the inclusive environment established in the metaverse shall be designed to be richer and more personal than the patient-therapist relationship established merely through conversations and words. By choosing different *experience settings*, patients going through the metaverse therapeutic process will then have a rich choice of schema modes for adapting to their living environment (Figure 2B). Their behavior in the real world will also be enriched by the set of healthy schema modes established in the metaverse that allow for transformative attempts without a great deal of willful effort. By breaking down solidified schema modes and establishing new ones, new behaviors will emerge more naturally and smoothly.

### Detailed process of the metaverse schema therapy toolbox

Let us consider the clinical case of 36-year-old Linda^1^ who had suffered from borderline personality disorders for long possibly due to her early experiences with her unattentative parents who had basically ignored her every need for care and love, and unfortunately, her attachment needs had not been satisfied throughout her life. Therefore, she was suffering from “strong mood swings, fits of anger, agitation, central insomnia characterized by waking up frequently and anhedonia,” which symptoms had turned worse after the leaving of her boyfriend who had stayed with her for 19 years. The traditional schema therapy would go through a “schema mode validation and education—limited reparenting with chair work and imagery rescripting—the Healthy Adult mode consolidation and integration” process, realized in close relationship with the therapist.

The metaverse schema therapy toolbox, in turn, would start from evaluating Linda’s relationship with the object world, then, instead of building a relationship with the therapist, the focus would be on building a relationship with the object world in the metaverse. Firstly, Linda will meet with a lovable virtual assistant who will guide her through the process. Then she will be asked to construct an important figure (e.g., her significant other) as her source of mental power; if it is not possible to construct, Linda could select a non-player character that best match her needs for safety and care. This figure will act as the model for the Healthy Adult mode and will finally be incorporated into Linda’s inner self. Then Linda will be invited to describe the problematic situation that is troubling her, in as much detail as possible, and construct the scenario described by Linda through natural language processing and biological feedback. Through validation of the constructed scenarios that Linda can see and experience in the metaverse, the early maladaptive schemas of Linda will be diagnosed and her schema modes will be categorized. The remote therapist at the background of the program will then prescribe several *experience settings* that aim at working with specific schema modes of Linda. For example, if the aim is to remove Linda’s Punitive Parent mode and care for the Abandoned and Abused Child mode, then Linda will be guided to enter a new world in the metaverse in which Linda will be embodied into her childhood avatar and interact with her virtual parents, though no longer punitive at all and instead will be attentive to her emotional needs; if the aim is to exchange the Detached Protector mode for the Healthy Adult mode, then Linda will be guided to enter another new world in the metaverse in which the problematic scene in her assessment stage will recur but with a totally different culture of feedbacks on her actions—she will be prompted to freely express her emotions and be welcomed with constructive feedbacks and love and care. Importantly, through different sets of experiences in the metaverse, Linda’s unmet emotional needs will be satisfied (as if it were all real) and her Healthy Adult mode will grow stronger and stronger, sometimes with the help from the abovementioned important figure to whom her secure attachment has been directed to if she feels her mental power is not strong enough to support her to enact behavior changes and emotional regulations. Ultimately, Linda will end her journey with successfully reshaped and enriched schema modes that will support her to better adapt to the real object world she is living in and even enact changes to her environment that becomes more inclusive to people like her.

### Ethical considerations

The aforementioned schema therapy process in the metaverse seems ideal, though there are several important ethical issues to be considered before application. Firstly, the respect of dignity and autonomy of the patient shall be protected in the metaverse. Secondly, the type of data collected from the patient needs to be communicated in advance (consent from the patient shall be acquired) and all collected data shall be firmly secured from leaking. Thirdly, the principle of “maximization of benefit and minimization of harm” shall be taken into full consideration when constructing scenarios and storylines, prescribing *experience settings*, and enacting interactions with the patient. Lessons shall also be learned from pioneers in the field. For example, Slater et al. (2020), Parsons (2021), and Chekroud et al., (2021) had all provided ethical guidance for virtual environment technologies.

## Reverse engineering: From the future to now

Now, where do we start from? There are two important missions from the reverse engineering perspective: the content and the technology.

For the content part, the critical mission is to accumulate an abundance of scenarios, storylines, figures, and contingencies for the accurate construction of therapeutic object world in the metaverse. Qualitative methods such as life-history interview and clinical case review could be used to find the key events and critical figures that can shape the behavioral patterns and change the schema modes in the life experiences of a representative sample of PD patients. Experimental work including animal behavioral modeling can be used to establish comparative models with key contextual factors influencing schema development. Formative materials can be developed, starting with interactive texts, moving to interactive videos and audios, then to the extended reality stage, and finally to the metaverse stage. Progressive monitoring is necessary to ensure the effectiveness of content updates and efficacy of interventions.

For the technology part, the *Minimal Viable Product* design method can be adopted: for proof of concept, one can simply start with the abovementioned interactive text toolbox, validate the design concept and treatment effects, and then upgrade the toolbox with feedbacks from the therapist, the patient, and the technological progress. Interfering issues such as cybersickness and burdened cognitive loads by the imperfect technology can be avoided by adopting this product design principle.

The overall efforts shall start from the preclinical phases to Phase I, II, and III just like other kinds of therapeutic tool development, with open minds to incorporate the discovery of new schema modes, new efficacious pathways, and theory advancement during the research and validation process.

To sum up, in addition to socio-economic considerations, PD patients usually interact with the object world in a way that both they and their counterparts feel uncomfortable, which can severely deter their minds to seek help from trusted mental health practitioners or have difficulty practicing what they have just learned from the therapist. The metaverse schema therapy toolbox could potentially help achieve adjustment and improvement at an early stage of PD symptoms, expand the beneficiaries of digital mental health services, reduce the stigma and the discomfort of some patients facing specific therapists, make it easier for people in need to move forward seeking help, and reduce the socio-economic costs of mental health services. It could also serve as a therapeutic tool for PD patients resistant to conventional therapies.

Just as Cieślik et al. (2020) quoted, “whatever limits the real world imposes on us, the virtual world is its ideal, unlimited reflection and creates a space where the impossible becomes possible, where modern technological solutions generate a new reality.”

## Data availability statement

The original contributions presented in the study are included in the article/supplementary material, further inquiries can be directed to the corresponding authors.

## Author contributions

BY and KJ conceived of the presented idea and critically revised the manuscript. BY, Y-XW, and C-YF wrote the first draft of the manuscript. All authors contributed to the article and approved the submitted version.

## Funding

This work was funded by the Natural Science Foundation of China (Project no. U1805263).

## Conflict of interest

The authors declare that the research was conducted in the absence of any commercial or financial relationships that could be construed as a potential conflict of interest.

## Publisher’s note

All claims expressed in this article are solely those of the authors and do not necessarily represent those of their affiliated organizations, or those of the publisher, the editors and the reviewers. Any product that may be evaluated in this article, or claim that may be made by its manufacturer, is not guaranteed or endorsed by the publisher.

## Footnotes

^1^This is the case described in Dadomo et al. (2016) as an example of applying the schema therapy to treat emotional dysregulations in borderline personality disorders.
